# Perovskite-Based Memristor with 50-Fold Switchable Photosensitivity for In-Sensor Computing Neural Network

**DOI:** 10.3390/nano12132217

**Published:** 2022-06-28

**Authors:** Qilai Chen, Tingting Han, Jianmin Zeng, Zhilong He, Yulin Liu, Jinglin Sun, Minghua Tang, Zhang Zhang, Pingqi Gao, Gang Liu

**Affiliations:** 1 School of Materials, Sun Yat-Sen University, Guangzhou 510275, China; chenqlai@mail.sysu.edu.cn (Q.C.); gaopq3@mail.sysu.edu.cn (P.G.); 2 Department of Micro and Nano Electronics, School of Electronic Information and Electrical Engineering, Shanghai Jiao Tong University, Shanghai 200240, China; hantingt1997@163.com (T.H.); jamy3531@sjtu.edu.cn (J.Z.); zhilong_he@sjtu.edu.cn (Z.H.); ldsunjinglin@163.com (J.S.); 3 School of Microelectronics, Hefei University of Technology, Hefei 230601, China; zhangzhang@hfut.edu.cn; 4 School of Materials Science and Engineering, Xiangtan University, Xiangtan 411105, China; ylliuxtu@163.com (Y.L.); tangminghua@xtu.edu.cn (M.T.)

**Keywords:** memristor, perovskite, machine vision, photosensitivity, neural network, in-sensor computing

## Abstract

In-sensor computing can simultaneously output image information and recognition results through in-situ visual signal processing, which can greatly improve the efficiency of machine vision. However, in-sensor computing is challenging due to the requirement to controllably adjust the sensor’s photosensitivity. Herein, it is demonstrated a ternary cationic halide Cs_0.05_FA_0.81_MA_0.14_ Pb(I_0.85_Br_0.15_)_3_ (CsFAMA) perovskite, whose External quantum efficiency (EQE) value is above 80% in the entire visible region (400–750 nm), and peak responsibility value at 750 nm reaches 0.45 A/W. In addition, the device can achieve a 50-fold enhancement of the photoresponsibility under the same illumination by adjusting the internal ion migration and readout voltage. A proof-of-concept visually enhanced neural network system is demonstrated through the switchable photosensitivity of the perovskite sensor array, which can simultaneously optimize imaging and recognition results and improve object recognition accuracy by 17% in low-light environments.

## 1. Introduction

As an essential branch of artificial intelligence (AI) technology, intelligent machine vision has been extensively applied in scientific, industrial, and consumer markets and has produced giant economic efficiency [1,2,3]. Traditional von Neumann architecture computers’ processing and memory blocks are physically separated [4,5]. When processing high-throughput computing tasks, separate memory and computing modules will frequently access and read data. Due to the different operating frequencies of the computing modules and memory modules, this process reduces the speed of processing tasks and increases energy consumption [6,7]. These drawbacks are particularly prominent in dealing with machine vision tasks because the conversion of image or video signals is accompanied by a large amount of data transmission and calculation [8].

Bioinspired neuromorphic in-memory or in-sensor digital/analog computing engineering, with high energy efficiency and low energy consumption, endow them as a potential candidate computing solution [9]. Modern image sensors on the basis of solid-state semiconductor technology could dependably capture the optical information thanks to the predefined photoresponsivity and internal chemical profile of silica-based materials [10,11]. Unfortunately, the fixed photoresponsivity makes it impossible to implement in-situ digital/analog computations in sensors. Therefore, traditional complementary metal-oxide-semiconductor (CMOS) image sensors require complex peripheral circuits to realize the in-sensor computing, which is a massive challenge for the manufacturing process. At the same time, the fill factor of the sensor decreases rapidly, as the physical space of the photosensitive area is taken up by the circuit, which makes the silicon-based device lose the advantages of high-fidelity imaging. Meanwhile, it dramatically reduces the imaging quality because of the circuit occupying the physical space of the photosensitive area [12].

The new principle device with intrinsic and tunable photoelectric properties displays a better development prospect in the near- and in-sensor bioinspired computing [13,14]. Interestingly, memristors with non-volatile and adjustable resistance and photoresponsivity could enable massively parallel sensing, storage, and computation with low power consumption [15]. In view of that, photosensitive memristors are expected to break through the bottleneck of traditional CMOS image processing technology, thereby improving the energy efficiency of the vision system while avoiding ineffective data transmission [16]. More significantly, the development of photoelectric memristors and integrated circuit requires the synchronous promotion of the intelligent materials, device structure, and architecture.

The materials of photosensitive memristors mainly include oxide materials [17], photochromic materials [18], perovskite materials [19], and two-dimensional materials [20]. Among them, organometallic halide perovskites have obvious advantages of large spectral absorption range, excellent charge separation and transfer, and superior photoresponsivity [21]. The broadband visible light response of perovskite permits the true-color imaging of examined targets, while the electric-field-induced ion migration of perovskites endows the non-volatile reconfiguration of internal potential profile and materials composition and further provides switchable photovoltaic responsivity. Therefore, perovskite-based device arrays can simultaneously possess high-fidelity adaptive imaging and real-time visual information processing.

From the device aspect of view, two main types of devices can realize the above-mentioned functions. One is a three-terminal sensor-computing integrated device with gate control, which can usually achieve more state control and higher amplitude control. However, the complexity and power consumption of three-terminal devices are relatively high [22,23]. The other is a sandwich-like two-terminal device that adopts the ion migration to regulate the photoresponsivity, while fewer adjustable states and range make it difficult to meet the higher-precision computing requirements [24]. Therefore, more in-depth research is required at the material and device structure levels to enhance the reliability, polymorphic regulation, and linearity of memristors, in order to promote the application of photoelectric memristors in the near- and in-sensor bioinspired computing.

This study presents a ternary cationic halide Cs_0.05_FA_0.81_MA_0.14_ Pb(I_0.85_Br_0.15_)_3_ (CsFAMA) perovskite in-sensor computing device that exhibits full-visible-spectrum photoresponse behavior and reconfigurable photosensitivity behavior. The external quantum efficiency (EQE) value is above 80% in the entire visible region (400–750 nm), and the peak responsibility value at 750 nm reaches 0.45 A/W. In order to solve the problem that the memristor photoresponse has a small adjustable range and cannot be maintained, we propose a hybrid modulation method of ion migration and readout bias. With this method, the tuning range can be over 50 times while the photoresponsivity is non-volatile. In addition, a proof-of-concept visually enhanced neural network system is demonstrated through the switchable photosensitivity of the perovskite sensor array, which can simultaneously optimize imaging and recognition results and improve object recognition accuracy by 17% in low-light environments. The visually enhanced neural network of in-sensor computing provides a promising strategy for future machine vision with excellent fidelity and efficiency.

## 2. Experimental Section

### 2.1. Materials Synthesis and Characterization

Methylammonium iodide and formamidine iodide (FAI, 99.999%) were provided by Greatcell Solar company. CsI (99.999%), PbI_2_ (99.999%), PbBr_2_ (99.999%), N, N-dimethylformamide (DMF, 99.8%), dimethyl sulfoxide (DMSO, 99.8%), and chlorobenzene (CB, 99%) were purchased from Sigma-Aldrich (St. Louis, MO, USA). Perovskite device based on CsFAMA perovskite film was fabricated with a configuration of ITO/CsFAMA/Au. The pre-patterned indium tin oxide (ITO) substrates were completely cleaned in deionized water, acetone, and isopropanol for 15 min, respectively. The CsFAMA precursor perovskite solution with a concentration of 1.4 mol/L was prepared in nitrogen-filled glovebox through dissolving 0.07 mol CsI, 1.134 mol FAI, 0.196 mol MAI, 1.085 mol PbI_2_, and 0.315 mol PbBr_2_ in DMF:DMSO = 4:1 (volume ratio) and stirred for 12 h. Subsequently, the substrate was treated with 15 min UV-ozone in plasma cleaner and ready for perovskite preparation. Afterward, 40 μL precursor solution filtered using 0.45 μm polytetrafluorethylene (PTFE) syringe filter was spin-coated to UV-treated ITO with the speed of 1000 and 4000 rpm for 10 s and 60 s, respectively. During the last 30 s of the spinning process, the substrate was treated by 180 μL anti-solvent chlorobenzene. Subsequently, the as-prepared layer was further annealed at 100 °C for 20 min, forming the final perovskite film.

### 2.2. Device Fabrication and Mensuration

ITO/CsFAMA/Au sandwich-like devices were acquired through depositing round-shape Aurum (Au) electrode through radio-frequency magneto sputtering with a metal shadow mask (TRP-450 system, Shenyang, China). The thickness and diameter of gold electrodes are 150 nm and 100 μm. The current–voltage curves of the devices were measured using a Keithley 4200 (Cleveland, OH, USA) semiconductor parameter analyzer with a vacuum probe station. Commercially LED lamps with white, blue (405 nm), green (532 nm), and red (655 nm) emissions were utilized as the light sources. The illuminating parameters can be controlled via a DORI RH-D12 single-chip microcontroller and a RXN3505M DC power supply unit, and calibrated through a Li-250A Light Meter (LI-COR, Inc., Lincoln, NE, USA). All electrical and optoelectronic measurements were carried out in the dark chamber of probe station to exclude the influence of the environment lights.

### 2.3. The Image Preprocessing Method

We randomly select a picture from the labeled faces in the wild home (LFW) face database as a demonstration. We convert the image from RGB to a single-pass grayscale image through OpenCV. At the same time, the photoresponse current of 0.05–12.75 nA was linearly mapped to the gray value of 0–255. Since the real response current will exceed the predetermined range, Python is used to define the value less than 0.05 nA as “0” and the value greater than 12.75 nA as “255”. The incident light intensity corresponding to each pixel can be converted by the photocurrent, and then we reduce the overall light intensity by 50% to simulate a weak light environment. Then, demonstrate the imaging effects of different light sensitivities in a low-light environment.

### 2.4. The Neural Network Construction Method

The function simulation in this work is implemented in the Python 3.6 environment. The databases used include TensorFlow, keras, Numpy, OpenCV, and Matplotlib. Using TensorFlow 2.5 version to build a single-layer perceptual network, we convert the image from RGB format to grayscale format through OpenCV. The relationship between light intensity and photocurrent response is mapped by Numpy. The Fashion-MNIST dataset is extracted from the Keras library [25]. The generation of pictures in low light is mainly realized by Numpy and Matplotlib. The visualization of neural network calculation results is achieved by TensorFlow and Matplotlib.

## 3. Results and Discussion

Considering the need for intrinsic device properties for in-sensor computing, we propose an organometal halide perovskite structure with reconfigurable ion migration and tunable photoelectric activity. Perovskites inherit the ABX_3_ structure of their inorganic components, allowing superior flexibility for compositional design. Moreover, the corner-sharing BX_6_ octahedra form an extended anionic framework, while the A site organic cations occupy the central cavity space [26,27,28]. The ionic radius and electronegativity of the cations give rise to a certain tilt in the crystal structure, which leads to changes in the band structure and lattice symmetry of perovskites [29,30,31]. Additionally, the incorporation of organic formamidinium (FA^+^) or methylammonium (MA^+^) monovalent cations, and the weak interaction with iodide anions, create indirect band gaps in perovskites. It broadens the light absorption range of perovskite materials [32,33,34,35].

**Figure 1:**
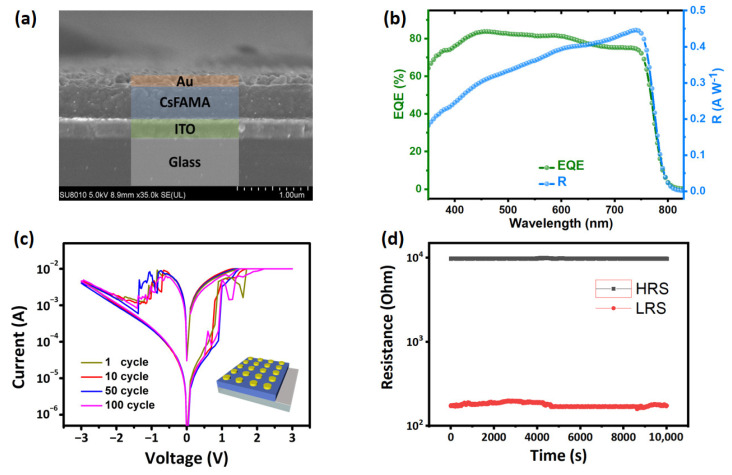
(**a**) Cross-section scanning electron microscope (SEM) image of ITO/CsFAMA/Au memristor; (**b**) external quantum efficiency (EQE) spectra and photoresponsivity (R) of CsFAMA-based devices; (**c**) DC I–V characteristics of the ITO/CsFAMA/Au memristor displaying switching behavior and the inset shows the structure of the device array; (**d**) room-temperature retention performance of the ITO/CsFAMA/Au memristor.

As shown in Figure 1a, the cross-section scanning electron microscope (SEM) shows that the devices of ITO/perovskite/Au were successfully fabricated with thicknesses of 150 nm, 300 nm, and 500 nm, respectively (Specific details are presented in Experimental Section). The EQE spectrum of CsFAMA-based photodetector was measured to acquire the photoresponse performance [36]. The EQE spectrum ranging from 350 to 850 nm was measured using a QE-R-Model of Enli Technology (Shanghai, China), and the light intensity at each wavelength was calibrated by a standard single-crystal Si cell. As depicted in Figure 1b, the EQE value is above or close to 80% in the entire visible region (400–750 nm), indicating the efficacious photon-to-current conversion rate over a broad spectral scope. More significantly, the responsibility (R) of CsFAMA device could be acquired from the EQE spectra by the following:

(1) R=EQE×λhce=EQE×λ1240 (A/W)

where λ and c is the wavelength and light speed, e is electron charge, and h is the Plank constant. Interestingly, the excellent responsiveness of the CsFAMA device to incident light, with its peak responsibility value at 750 nm reaching 0.45 A/W, is comparable to that of the existing silicon-based detectors. The schematic diagram of overall device structure is depicted in the inset of Figure 1c. In the DC swept voltage mode, the device displays evident bipolar resistive switching behavior and a storage window close to two orders of magnitude, demonstrating a device’s high plasticity in polymorphic modulation (Figure 1c). Two stable resistance states (high resistance state (HRS) and low resistance state (LRS)) can be acquired by regulating the limiting current during the set process and the cut-off voltage during the reset process. When the applied voltage rises from 0 V to 3 V, the resistance will change from HRS to LRS. When applying voltage rises from 0 V to −3 V, the device will also change from LRS to HRS. It is worth noting that the electrical stimulation will not change the existing state of the device as the operating voltage is within −0.5 V to 0.5 V, which proves that the bias voltage can enhance the photocurrent without changing the existing state of the device. At the same time, the retention test of the device at room temperature suggests good stability in both high and low resistance states (Figure 1d).

**Figure 2:**
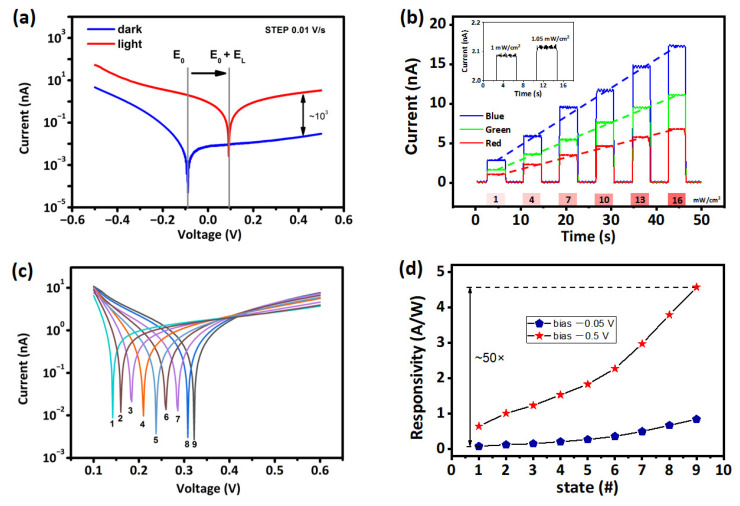
(**a**) Current–voltage characteristic curve under 16 mW/cm^2^ light and dark state, respectively; (**b**) photocurrent responses of ITO/CsFAMA/Au device read at 0.1 V, under blue (405 nm), green (532 nm), and red (655 nm) illuminations with different optical powers for 5 s in the inset picture, the photoresponse of the device is shown at 1.00 mW/cm^2^ and 1.05 mW/cm^2^, respectively; (**c**) photovoltaic characteristics and (**d**) photoresponsitivies of the Cs_0.05_FA_0.81_MA_0.14_ Pb(I_0.85_Br_0.15_)_3_ (CsFAMA) device obtained under white light irradiation of 16 mW/cm^2^, after being subjected to 1 V constant voltage stressing at for different periods (1–9 represent different bias stimulation times ranging from 0 s to 80 s in 10 s increments), respectively.

Moreover, the influence of illumination on the current–voltage curve of device is displayed in Figure 2a. As the bias voltage is scanned from 0.2 to 0.5 V, the light-dark ratio is close to 10^3^, whereas the light-dark ratio is greater than 30 during scanning from −0.2 V to −0.5 V. Moreover, the photoelectric ratio detection rate and light-dark ratio are positively correlated parameters, and a higher light-dark ratio provides the hardware basis for high-definition imaging. Consistent with the full visible spectral absorption behavior, the ITO/CsFAMA/Au device exhibits sensitive photoresponsivity to blue (405 nm), green (532 nm), and red (655 nm) illumination. As depicted in Figure 2b, with the increment of optical power from 1 to 16 mW/cm^2^, excellent linearity of photocurrent (read at −0.5 V) versus light density could be acquired for all RGB stimulations. From the inset of Figure 2b, it can be observed that the photoresponse current can still distinguish the devices with the 0.05 mW/cm^2^ increment of optical power density. In view of that, a single-pixel incorporating three CsFAMA photovoltaic devices for individual RGB sensing can detect 16 million combinational colors for true-color imaging.

In perovskite-based two-terminal devices, the photoresponsivity can be controlled non-volatilely by changing the ionic state inside the devices [37,38]. According to the resistance-to-transfer curve of the device in Figure 1c, the device internal will undergo significant ions migration resulting in the resistance state change when the bias voltage exceeds 1 V. As such, the extensive ion migration range is beneficial for storage. However, a significant resistance change is not allowed in the modulation of photosensitivity because the resistance change would adversely lead to the increase of dark current. Therefore, the modulation voltage is set at 1 V, which can ensure the migration of ions in the device, but not very fast and violent.

More significantly, the ions with low activation energy in perovskite such as monovalent organic cation (MA^+^, FA^+^) and halogen anion (Br^−^, I^−^) have appropriate diffusion/drift coefficient and mobility. By applying the bias voltage, the ions migration would change the chemical composition and generate an additional ion field to finely tune the photovoltaic characteristics. As shown in Figure 2c, after each bias stimulation with the increment of 10 s, current–voltage characteristics were tested under the illumination of 16 mW/cm^2^. Significantly, the relationship between photovoltaic properties and photocurrent responses of the devices changes regularly with the duration of the electrical stimulation. Due to the logarithmic coordinate used for the ordinate, the current value takes the absolute value. The actual current value is positive to the right and negative to the left of the lowest point. As the modulation bias duration increases, the current shifts to the right, and the photoresponse current increases. It means that the ion migration results in changes in the photovoltaic and photoconductivity performance of the device. Therefore, the photoresponsivity of the device could be regulated by applying a bias greater than 1 V. The adjustable photoresponsivity device can operate a simple multiplication by using the “light intensity”, “photoresponsivity”, and “response current” as “input”, “weight”, and “output”, respectively. However, it is still difficult to realize the modulation of more photoresponsivity states through ion migration because the change of dark current will affect the accuracy of the photoresponsivity calculation. We propose a hybrid modulation approach that modulates the device photoresponsivity by combining varying readout voltages and modulating ion mobility. Specifically, we employed ion mobility to achieve nonvolatile photosensitivity modulation, and then enhance the photosensitivity of the device by increasing the readout voltage. As shown in Figure 2d, the ion migration within the device can tune the photoresponsivity of the device. When the readout voltage is −0.05 V, the photoresponsivity control range is between 0.08 A/W and 0.82 A/W. When the readout voltage is −0.5 V, the photoresponsivity control range is between 0.61 A/W and 4.66 A/W. With a fixed readout voltage, the ion migration within the device can increase photoresponsivity about 10-fold. Alternatively, the photoresponsivity can be improved by a factor of about 50 through hybrid modulation of ion mobility and readout voltage. Considering the linearity of the photoresponsivity and the conditions of ion migration, the readout voltage cannot be increased indefinitely. Here we set the maximum read bias to −0.5 V. Because it is easy to realize the multi-level regulation of the bias voltage by using the digital to analog converter (DAC) module, the hybrid modulation method not only increases the control range of the photoresponsivity of the device but also increases the number of states of the photoresponsivity [39,40,41]. With regard to photoresponsivity as the weight of the neural network, the use of hybrid modulation can easily make the accuracy of the weight reach 8 bits, which can further endow the in-sensor computing with more accurate results.

For perovskite thin films, exposure to optical illumination directly generates the bound excitons that further dissociate into free holes and electrons due to its small bandgap [42,43]. The built-in potential barrier or the external bias voltage can make the free electrons and holes move directionally to the electrode to generate the photocurrent. Therefore, adjusting the built-in potential barrier or the external bias voltage is the two main ways to adjust the photoresponsivity [44].

**Figure 3:**
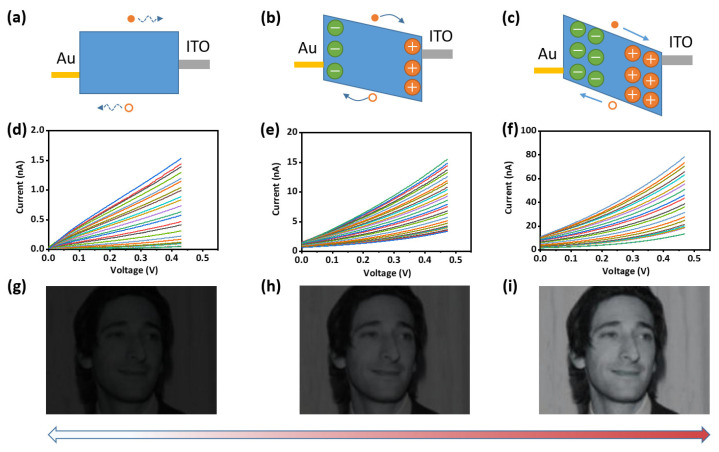
(**a**–**c**) Schematic diagram of energy band structure in different ion distribution states of perovskite; (**d**–**f**) I–V curves of the ITO/CsFAMA/Au photodetector under white light irradiation with different optical intensity through applying 1 V bias for (**d**) 0 s; (**e**) 40 s; (**f**) 80 s; (**g**–**i**) imaging simulation results of CsFAMA photosensitive array under different photoresponsivity. This figure is from labeled faces in the wild home (LFW) (http://vis-www.cs.umass.edu/lfw/, accessed on 20 March 2022).

To clearly understand the mechanism of the control of photoresponsivity, a schematic diagram was depicted in Figure 3a–c. As the initial state of the two-terminal device, photoexcited free electrons and holes do not migrate directionally due to the absence of electron and hole transport layers. In this state, the bias voltage dominates the direction of free electron and hole migration. Driven by a larger bias voltage, the charge carriers would migrate to the anode and cathode, respectively, and generate a built-in ionic field of opposite polarity to the bias voltage (Figure 3b). After removing the bias voltage, most of the ions will stay in place due to the migration barrier of ions, thus preserving the ion field. Additionally, the migration of ions will inevitably lead to the generation of defects (e.g., interstitial defects and replacement defects) and results in the change of chemical compositions and these changes are irreversible even removing the bias voltage. Meanwhile, the photoexcited free electrons and holes can move directionally to generate current even without the bias. Therefore, the photoresponsivity of the device can be changed non-volatilely by a large bias voltage. More importantly, the duration of applying the large bias voltage can control the size of the ion field, thereby realizing the multi-level adjustment of photoresponsivity (Figure 3c). As displayed in Figure 3d–f, different photoresponsivity and I–V curves are obtained through applying 1 V bias voltage for 0, 40, and 80 s, respectively. As shown in Figure 3d, the photoresponse current of the photosensor is relatively low as a whole owing to no ion migration occurring, while the response current increases rather linearly with light intensity. Theoretically, the initial states with high linearity are best suited for light–input neuromorphic computations. As shown in Figure 3e, due to the ion field generated after 40 s bias being applied, the photoresponse current increases significantly under the same illumination and bias voltage, and the linearity of the current response curve begins to decrease. Moreover, the above phenomenon is further enhanced as more prolonged bias stimuli enhance the ion field (Figure 3f). The imaging quality of the device under different photoresponsivity was also simulated. Here, we artificially define the mapping relationship between current and image gray value under standard conditions to facilitate the visualization of functional simulations (Specific details are presented in the Experimental Section). The measured data from three devices with different photosensitivity are prepared as a look-up table relating bias voltage, light intensity, and response current. By extracting the light intensity and response current data of three devices, the imaging effects of different photosensitive devices under low light are simulated. To exclude the influence of bias voltage, the readout voltage is uniformly set to 0.2 V. It can be seen from Figure 3g–i that, in a low-light environment, the higher the intrinsic photoresponsivity of the device, the easier it is to obtain more explicit images and more details.

The traditional artificial visual system depends on the CMOS-based image sensor and a control system to convert the optical signal into voltage spikes [45,46,47]. Then, the data are transmitted to the computer for neural network function calculation. In the face of high-definition video tasks, data transmission will seriously affect the computing speed and increase power consumption [48]. Machine vision involving in-sensor computing enables faithful capturing of the visual information from the environment and provides in-situ efficient image processing capabilities, which can significantly reduce the data shuttle between the computing and sensor unit [49]. It is significant for practical user-end applications with fast responding and decision-making, such as instant detection and dodging of on-road objects during automatic drive.

**Figure 4:**
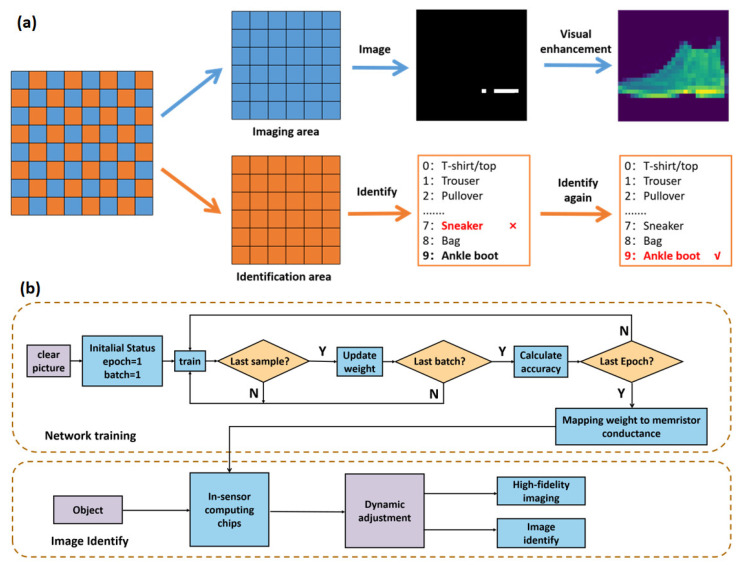
(**a**) Schematic diagram of the vision-enhanced in-sensor computing neural network; (**b**) schematic flowchart for the supervised learning simulation with artificial neural network for high-fidelity imaging and image identify.

As shown in Figure 4a, we propose an AI vision scheme that can simultaneously image and recognize dynamic accommodation. Two adjacent pixels of the image sensor are responsible for imaging (blue pixels) and computation (orange pixels), respectively. Although the division of labor is different, the device structure and preparation process are entirely the same, which is expected to reduce the process complexity of chip manufacturing. In terms of imaging, the device will not be subjected to a large bias voltage to ensure the consistency of photoresponsivity. In the weak light environment, the readout voltage can adjust the photoresponsivity to enhance imaging quality. In terms of in-sensor computing, the device photoresponsivity modulated by ion field is employed as the weight of a neural network. Compared with changing the photoresponsivity through readout voltage, this method can non-volatilely save the weight of the neural network and reduce the average power consumption of the entire sensor chip. Similarly, in the low-light environment, the readout voltage can be adjusted synchronously with the imaging unit to improve the image recognition accuracy. In this way, the in-sensor computing chip can dynamically adjust the imaging, recognition, and calculation results according to the light intensity of the environment.

Figure 4b shows the specific algorithm flow diagram. The skeleton of the network adopts a single-layer perceptual network, where the network weight bits are 7840 (28 × 28 × 10). Fashion-MNIST is employed as the training set and test set of the network, of which the number is 10,000 and 50,000, respectively [25]. Additionally, the back propagation algorithm is utilized for 10 epoch training to update the neural network weights [50]. When the computer performs neural network functions, the weights are updated and stored with 8-bit precision. However, for the realization of artificial neural network hardware algorithms, such high weights are difficult to achieve through precise voltage regulation [51,52]. Since the device can achieve more than eight photosensitive states, we adopt two components as the neural unit responsible for the positive and negative weights, respectively. In this way, the device finally can map 16 states. In the neural network, the weights will be quantized to 4-bit precision to simulate the working state of the in-memory computing chip in a more realistic manner.

**Figure 5:**
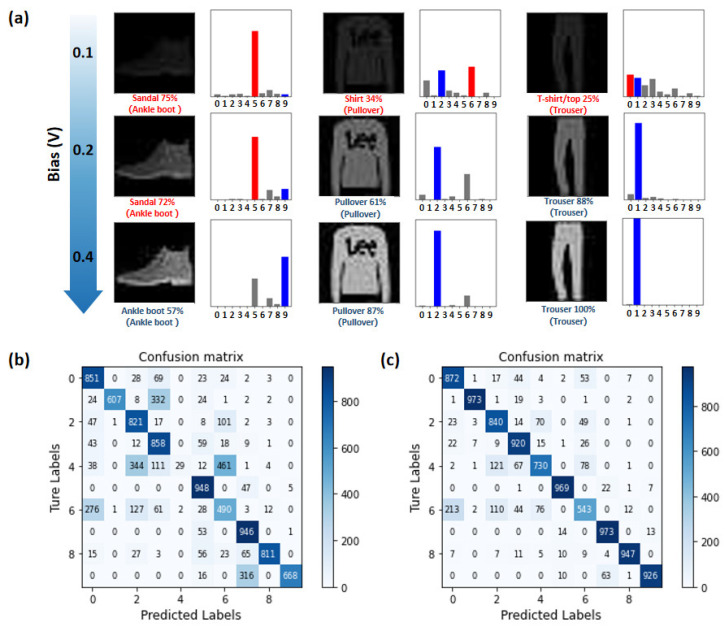
(**a**) The relationship between imaging results and recognition accuracy under different bias readout voltages. The images are adapted with permission from [25]; (**b**,**c**) confusion matrix diagram of the in-sensor computing neural network at 0.1 V and 0.4 V bias voltage, respectively.

Based on the mapping relationship constructed by the look-up table built in Figure 3d, a series of test images are generated to simulate the adjustment of the image in weak light by the bias voltage and the change of the recognition rate of the neural network (Specific details are presented in Experimental Section). As shown in Figure 5a, neither the imaging information nor the recognition results are ideal when the applied light is too weak. The neural network recognizes “Ankle boot”, “Pullover”, and “Trouser” as “Sandal”, “Shirt”, and “T-shirt”, respectively. When the readout voltage is 0.2 V, the image becomes more precise, and only “Ankle boot” is mistaken for “Sandal” in the above three pictures. All pictures can be accurately identified with the continuous increment of readout voltage to 0.4 V. To further explain the experimental results, the neural network’s confusion matrix of recognition results when the read bias is 0.1 V and 0.4 V have been shown in Figure 5b,c, respectively. Under the 0.1 V bias voltage, the overall recognition rate is 70%, while the recognition accuracy rate reaches 87% under 0.4 V bias voltage, which is pretty close to the theoretical highest recognition rate (92%) of a single-layer neural network. We believe that sensor-computing integrated devices based on perovskite materials can provide a promising pathway for implementing the proposed adaptive imaging and in-sensor neuromorphic pattern recognition missions.

## 4. Conclusions

This work presents a ternary cationic halide CsFAMA-based perovskite in-sensor computing device that displays full-visible-spectrum photoresponse capability and reconfigurable photosensitivity behavior. As expected, the experimental results show that the photoresponsivity of the device can be increased more than 50 times by adjusting the degree of internal ion migration and the setting of the readout bias. It is noted that the sensor can achieve high-definition imaging of the target object in low light by adjusting the overall light responsivity. In terms of in-sensor computing, simple computing tasks can be performed by independently adjusting the photoresponsivity of each element in the array. At the same time, the accuracy of neural network is significantly improved by adjusting the readout bias voltage under weak light. The in-sensor computing showcased in this contribution provides a promising strategy for future machine vision with excellent fidelity and overall efficiency.

## Data Availability

The raw data supporting the conclusions of this article will be made available by the authors, without undue reservation.
